# Black carbon content in airway macrophages is associated with increased severe exacerbations and worse COPD morbidity in SPIROMICS

**DOI:** 10.1186/s12931-022-02225-0

**Published:** 2022-11-14

**Authors:** Vickram Tejwani, Han Woo, Chen Liu, Anna K. Tillery, Amanda J. Gassett, Richard E. Kanner, Eric A. Hoffman, Fernando J. Martinez, Prescott G. Woodruff, R. Graham Barr, Ashraf Fawzy, Kirsten Koehler, Jeffrey L. Curtis, Christine M. Freeman, Christopher B. Cooper, Alejandro P. Comellas, Cheryl Pirozzi, Robert Paine, Donald Tashkin, Jerry A. Krishnan, Coralynn Sack, Nirupama Putcha, Laura M. Paulin, Marina Zusman, Joel D. Kaufman, Neil E. Alexis, Nadia N. Hansel

**Affiliations:** 1 Division of Pulmonary and Critical Care Medicine, Johns Hopkins University, Baltimore, MD USA; 2 Respiratory Institute, Cleveland Clinic, 9500 Euclid Avenue, A90, 44195 Cleveland, OH USA; 3 Center for Environmental Medicine, Asthma, and Lung Biology, Division of Allergy and Immunology, University of North Carolina at Chapel Hill, Chapel Hill, NC USA; 4 Division of Pulmonary and Critical Care Medicine, University of Washington, Seattle, WA USA; 5 Division of Respiratory, Critical Care and Occupational Medicine, University of Utah, Salt Lake City, UT USA; 6 Department of Radiology, Medicine and Biomedical Engineering, University of Iowa, Iowa City, IA USA; 7 Division of Pulmonology and Critical Care Medicine, Weill-Cornell Medical Center, Cornell University, New York, NY USA; 8 Division of Pulmonary, Critical Care and Sleep Medicine, University of California San Francisco, San Francisco, CA USA; 9 Department of Medicine, Columbia University College of Physicians and Surgeons, New York, New York, USA; 10 Environmental Health and Engineering, Johns Hopkins University, Baltimore, MD USA; 11 Pulmonary and Critical Care Medicine Division, Department of Internal Medicine, University of Michigan Health System, Ann Arbor, MI USA; 12 Veterans Affairs Ann Arbor Healthcare System, Ann Arbor, MI USA; 13 Division of Pulmonary and Critical Care Medicine, University of California Los Angeles Medical Center, Los Angeles, CA USA; 14 Division of Pulmonary, Critical Care, and Occupational Medicine, College of Medicine, University of Iowa, Iowa City, IA USA; 15 University of Utah Hospital, Salt Lake City, UT USA; 16 Department of Medicine, University of Illinois at Chicago, Chicago, IL USA; 17 Pulmonary/Critical Care, Dartmouth Hitchcock Medical Center, Lebanon, NH USA

**Keywords:** Macrophage, Black carbon, COPD

## Abstract

**Background:**

Airway macrophages (AM), crucial for the immune response in chronic obstructive pulmonary disease (COPD), are exposed to environmental particulate matter (PM), which they retain in their cytoplasm as black carbon (BC). However, whether AM BC accurately reflects environmental PM_2.5_ exposure, and can serve as a biomarker of COPD outcomes, is unknown.

**Methods:**

We analyzed induced sputum from participants at 7 of 12 sites SPIROMICS sites for AM BC content, which we related to exposures and to lung function and respiratory outcomes. Models were adjusted for batch (first vs. second), age, race (white vs. non-white), income (<$35,000, $35,000~$74,999, ≥$75,000, decline to answer), BMI, and use of long-acting beta-agonist/long-acting muscarinic antagonists, with sensitivity analysis performed with inclusion of urinary cotinine and lung function as covariates.

**Results:**

Of 324 participants, 143 were current smokers and 201 had spirometric-confirmed COPD. Modeled indoor fine (< 2.5 μm in aerodynamic diameter) particulate matter (PM_2.5_) and urinary cotinine were associated with higher AM BC. Other assessed indoor and ambient pollutant exposures were not associated with higher AM BC. Higher AM BC was associated with worse lung function and odds of severe exacerbation, as well as worse functional status, respiratory symptoms and quality of life.

**Conclusion:**

Indoor PM_2.5_ and cigarette smoke exposure may lead to increased AM BC deposition. Black carbon content in AMs is associated with worse COPD morbidity in current and former smokers, which remained after sensitivity analysis adjusting for cigarette smoke burden. Airway macrophage BC, which may alter macrophage function, could serve as a predictor of experiencing worse respiratory symptoms and impaired lung function.

**Supplementary Information:**

The online version contains supplementary material available at 10.1186/s12931-022-02225-0.

## Background

Chronic obstructive pulmonary disease (COPD) is characterized by recurrent exacerbations and progressive decline in lung function due in part to abnormal inflammatory responses of the airways to noxious particles or gases [1–3]. In developed countries, the most strongly supported cause of COPD is cigarette smoking; however, there is growing evidence that other environmental exposures, particularly ambient and indoor air pollution, are linked to cardiopulmonary morbidity and mortality [4, 5]. Inhaled fine (< 2.5 μm in aerodynamic diameter) particulate matter (PM_2.5_) reaches the surfaces of the central airways where airway macrophages (AM) reside. AM, which play a critical role in immune response and tissue repair in COPD, are exposed to particles which are phagocytosed and retained in the cytoplasmic material [6–8]. Both cigarette smoke [9] and the carbonaceous core that commonly comprises particulate matter PM_2.5_ can be visualized within AM [10]. Thus, AM black carbon (BC)–quantified as mean BC cross-sectional area (um^2^) per macrophage–may serve as a biomarker for airborne PM pollutant and tobacco smoke exposure. This has the potential to estimate prior exposures without requiring the burdensome and complex process of direct in-home measurements.

Hence, cytopathologic techniques that examine macrophages collected from induced sputum may have the ability to assess an individual’s inhaled dose of particles [11, 12]. In single center studies, inhaled smoke from combusted biomass and indoor PM have been linked to higher AM BC in healthy volunteers and former smokers with COPD, respectively [13, 14]. We also recently reported an association of AM BC area with increased severe exacerbations and nominally worse lung function among a small cohort of former smokers with COPD [15].

However, AM BC has previously been examined primarily in small studies in a single geographic location, limiting definitive conclusions regarding exposure contributions to AM BC content and its association with respiratory outcomes. Additionally, macrophages vary in size [16, 17] and therefore, the percent of macrophage occupied by black carbon rather than absolute area may be relevant – however, most studies did not assess AM BC percent area. We sought to assess the relationship between ambient and indoor pollutant exposures and AM BC (quantified as area and percent area) in former and current smokers from the Subpopulations and Intermediate Outcome Measures In COPD Study Air Pollution (SPIROMICS AIR) Study and to assess further the relationship of AM BC and COPD morbidity. We hypothesized that smoking and particulate exposure lead to AM BC which may lead to worse COPD morbidity given dysregulated macrophage function.

## Methods

### SPIROMICS-AIR cohort

SPIROMICS (ClinicalTrials.gov NCT01969344) is a prospective observational cohort study with the goals of identifying new COPD subgroups and intermediate markers of disease progression. SPIROMICS AIR added air pollution exposure assessment to estimate individual-level outdoor and indoor air pollution exposures in a subgroup of participants. Subjects for SPIROMICS AIR were enrolled from 2010 to 2016, phenotyped and followed at seven centers [18]. SPIROMICS participants were 40–80 years of age at baseline, who were either healthy persons with a smoking history of ≤ 1 pack-year (“never smokers”) and no known current lung disease or current or former smokers (≥ 20 pack-years) with and without evidence of obstructive lung disease. Given our focus on those with or at risk of COPD, never smokers were not included in this analysis. Informed written consent was obtained from all participants, all experiments were performed in accordance with relevant guidelines and regulations, and the study was approved by the IRB and ethics committee of each site **(e-Table 1)**.

We categorized participants as having either no spirometric evidence of airflow obstruction or having COPD (postbronchodilator FEV1 /FVC < 70%) with Global Initiative for Chronic Obstructive Lung Disease (GOLD) stage I/II (FEV1 ≥ 50% predicted) or GOLD stage III/IV (FEV1 < 50% predicted) disease. A concomitant asthma diagnosis was not an exclusion criterion, however participants were queried regarding a history or diagnosis of asthma. Those with other non-COPD obstructive lung disease were excluded.

### Airway macrophage black carbon assessment

Sputum induction was performed according to the methods of Alexis et al. [19]. Three hundred sixty-three subjects at SPIROMICS AIR sites with a post bronchodilator FEV_1_ > 35%, underwent sputum induction. Of these, 17 never smokers were excluded and 22 had slides that were poor quality or did not have an adequate number of macrophages leaving 324 subjects selected for BC analysis. Cytospin samples were prepared as outlined in the Online Supplement. A total of 50 AM, identified by morphology and with an intact cell wall irrespective of BC content, were randomly selected per participant. The 50 AM were analyzed for BC by a trained technician blinded to environmental exposure assessment and clinical outcomes. Processing slides in two batches, we quantified AM BC both as mean cross-sectional area (um^2^) per macrophage and as mean percent area BC per macrophage which is the cross-sectional area of BC divided by the cross-sectional area of the macrophage [14, 20].

### Exposure assessment

Estimation of ambient outdoor concentrations of PM_2.5_, nitrogen dioxide (NO_2_) and ozone outside of each participant’s residential addresses, which relied on spatio-temporal modelling as described in the Online Supplement, were available for 284 participants [21, 22]. For this analysis, ambient exposures were averaged over the one month prior to sputum induction and BC quantification.

Modeled indoor air pollutant concentration (PM_2.5_,NO_2,_ and nicotine) estimates were available for 170 participants, using models generated from SPIROMICS as previously described [23] and as described in the Online Supplement. When compared to the measured concentrations available on a subset of patients, the models explained 60%, 63% and 61% of variability in PM_2.5_, NO_2_ and indoor nicotine, respectively [23]. Urinary cotinine was also measured at the study visit using quantitative liquid chromatography-tandem mass spectrometry (ARUP Laboratories, Salt Lake City, UT). Current and former smokers were defined based on a urinary cotinine cutoff of 31 ng/mL which has been shown to have a sensitivity of 97.1% and specificity of 93.9% for active smoking [24]. We also specifically queried participants regarding exposures to vapors, gases, dusts, or fumes (VGDF).

### Spirometric and clinical assessment

Spirometry before and after bronchodilator was performed according to standard guidelines [25] and reviewed for quality control and assurance. Exacerbations were assessed prospectively for one-year by quarterly phone calls. We defined exacerbation as worsening respiratory symptoms requiring antibiotics or oral steroids, and severe exacerbations as leading to an emergency department visit or hospitalization.

On the same day as sputum collection, health information was collected through validated questionnaires; specifically, the St. George’s Respiratory Questionnaire (SGRQ), [26] COPD Assessment Test (CAT), [27] the modified Medical Research Council (mMRC) Dyspnea Scale [28] and ease of cough and sputum questionnaire [29]. We defined chronic bronchitis by a positive response to either the classic or alternative SGRQ definition [30]. Functional exercise capacity was assessed by the 6 min walk distance (6MWD), performed according to American Thoracic Society criteria [31].

### Statistical analysis

We used descriptive statistics to characterize sample participants with AM BC data and compare their characteristics to the larger SPIROMICS cohort.

To assess the relationship between AM BC and indoor PM_2.5_, we ran a linear regression of BC area or BC percent on indoor PM_2.5_ using generalized linear mixed model with random effect for study sites. The models were minimally adjusted for batch (first vs. second) and fully adjusted for batch, age, race, income, BMI, and long-acting beta-agonist or long-acting muscarinic antagonist (LABA/LAMA) use (see **Online Supplement** for additional detail). Pack-years was considered as a covariate but given it was not associated with either AM BC percent or area, we did not include it in our model. Sensitivity analysis was conducted using urinary cotinine as a covariate in the fully adjusted models. As secondary analysis, other environmental exposures (indoor NO_2_, indoor nicotine, urinary cotinine, VGDF exposure (yes/no), ambient PM_2.5_, ambient NO_2_, and ambient ozone) were assessed for their associations with BC area or BC percent one at a time using the same regression approach.

To assess the relationship between COPD outcomes and AM BC, linear regression for continuous COPD outcomes and logistic regression of any or severe exacerbations and chronic bronchitis was run on BC area or BC percent using a generalized linear mixed model with random effect for study sites. All models were adjusted for batch, age, sex, race, income, BMI, and LABA/LAMA use (see Online Supplement). For severe exacerbation model only, due to low number of reported events, the covariates were limited to batch without random site effect. In sensitivity analysis, urinary cotinine and FEV1% predicted were additionally adjusted as a covariate one at a time. To assess whether the association between COPD outcomes and AM BC varied by smoking status, a two-way interaction model was run by adding the interaction between dichotomous smoking status (> or ≤ 31.5 ng/mL urinary cotinine level [24]) and BC area or BC percent to the fully adjusted model. Given cotinine can be influenced by nicotine replacement therapy, analyses were also repeated by self-reported smoking status. Additionally, stratified analyses by smoking status were performed (see Online Supplement). For all regression analyses, linearity and normality assumptions were checked where appropriate and transformations made as needed (see Online Supplement).

All analyses were completed using STATA version 15.1 (College Station, TX). Statistical significance was defined as P < 0.05 for main and interaction effect.

## Results

### Participant characteristics

Participants had a mean (SD) age of 63.6 (8.8) years (Table 1). 41% of the participants were female, 81% were white, and 69% had education beyond high school. About half of the participants (44%) self-reported as current smokers, but 160 (50%) had urinary cotinine > 31.5 ng/ml. Of self-reported current smokers, 99% had a urinary cotinine > 31.5ng/mL, whereas 89% of self-reported former smokers had a urinary cotinine < 31.5 ng/mL. In the year prior to baseline visit, 16.1% and 6.9% of the participants reported any and severe exacerbations, respectively.

**Table 1:** Participant Characteristics, N = 324

	Mean ± SD or n (%)
Age, yrs	63.58 ± 8.78
Sex, n (% female)	134 (41.4%)
Race, n (% white)	260 (80.7%)
Education, n (% > HS grad)	223 (69.0%)
Annual Household Income	
<$35,000	116 (35.8%)
$35,000 - $74,999	101 (31.2%)
≥$75,000	62 (19.1%)
Decline to answer	45 (13.9%)
Body Mass Index, kg/m^2^	28.50 ± 5.07
Medication LABA/LAMA, n (%)	87 (27.1%)
Smoking (pack-years)	47.76 ± 22.46
FEV_1_, percent predicted	84.30 ± 18.96
Current Smoker, n (%)	143 (44.4%)
Urinary Cotinine, ng/mL	597.70 ± 851.23
VGDF, n (%)	142 (44.2%)
ICS, n (%)	69 (21.5%)
OCS, n (%)	2 (0.6%)
CAT score	12.31 ± 7.72
mMRC score	0.83 ± 0.82
SGRQ score	28.26 ± 19.34
ECSC	9.16 ± 3.49
6MWD, meters	432.99 ± 101.29
Any exacerbations in the prior 12mo, n (%)	51 (16.1%)
Severe exacerbations in the prior 12mo, n (%)	22 (6.9%)
Any exacerbations over 12mo follow-up, n (%)	36 (11.9%)
Severe exacerbations over 12mo follow-up, n (%)	8 (2.6%)
Sputum Cell Differential	
Neutrophil %	59.81 ± 21.77
Macrophage %	37.37 ± 20.65
Eosinophils %	0.98 ± 2.27
Lymphocytes %	0.11 ± 0.24
Basophils %	0.00 ± 0.03
Bronchial epithelial cells %	1.94 ± 3.69
Asthma	
Never had asthma, n (%)	243 (75.0%)
Diagnosed with asthma, n (%)	61 (18.9%)
Don’t know if had asthma, n (%)	20 (6.2%)

**Fig. 1:**
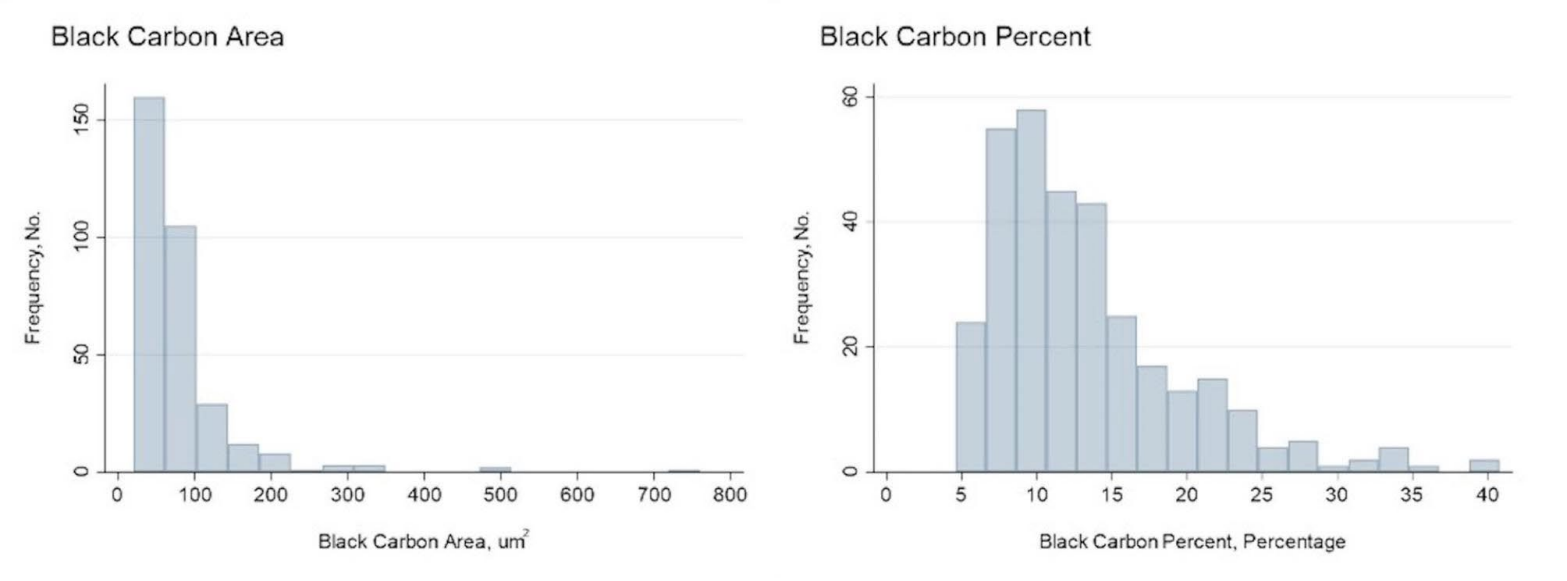
Histograms showing distribution of black carbon area and black carbon percent

The mean (SD) BC area was 80.68 (70.56) µm^2^ [median (Q1-Q3) = 61.7 (44.8–87.8)] and BC percent 13.29 (6.45)% [median (Q1-Q3) = 11.5 (8.7–16.0)](Fig. 1**and e-Figs. 1 and 2**). The participants in our analysis had less severe disease but a similar pack-year history and exposure burden, compared to all participants in the SPIROMICS cohort with an FEV_1_ > 35% predicted **(e-Table 2)**.

### Environmental exposure association with macrophage black carbon

Two hundred eighty-five (88%) participants had modeled indoor and ambient pollutant estimates. The mean (SD) modeled indoor PM_2.5_ and NO_2_ concentration were 12.00 (10.16) µg/m^3^ and 11.44 (4.86) ppb, respectively. Modeled indoor airborne nicotine had a mean (SD) of 33.96 (22.28) µg/m^3^, and 142 (44.2%) reported vapor dust gas fume (VDGF) exposure. The modeled mean (SD) ambient PM_2.5_, NO_2_, and ozone concentration over the month prior to quantification were 8.75 (2.95) µg/m^3^, 11.11 (7.35) ppb, and 26.14 (8.23) ppb respectively. There was generally no difference in participant characteristics between those with and without estimates of air pollutant exposure except for education and income level **(e-Table 3)**.

**Fig. 2:**
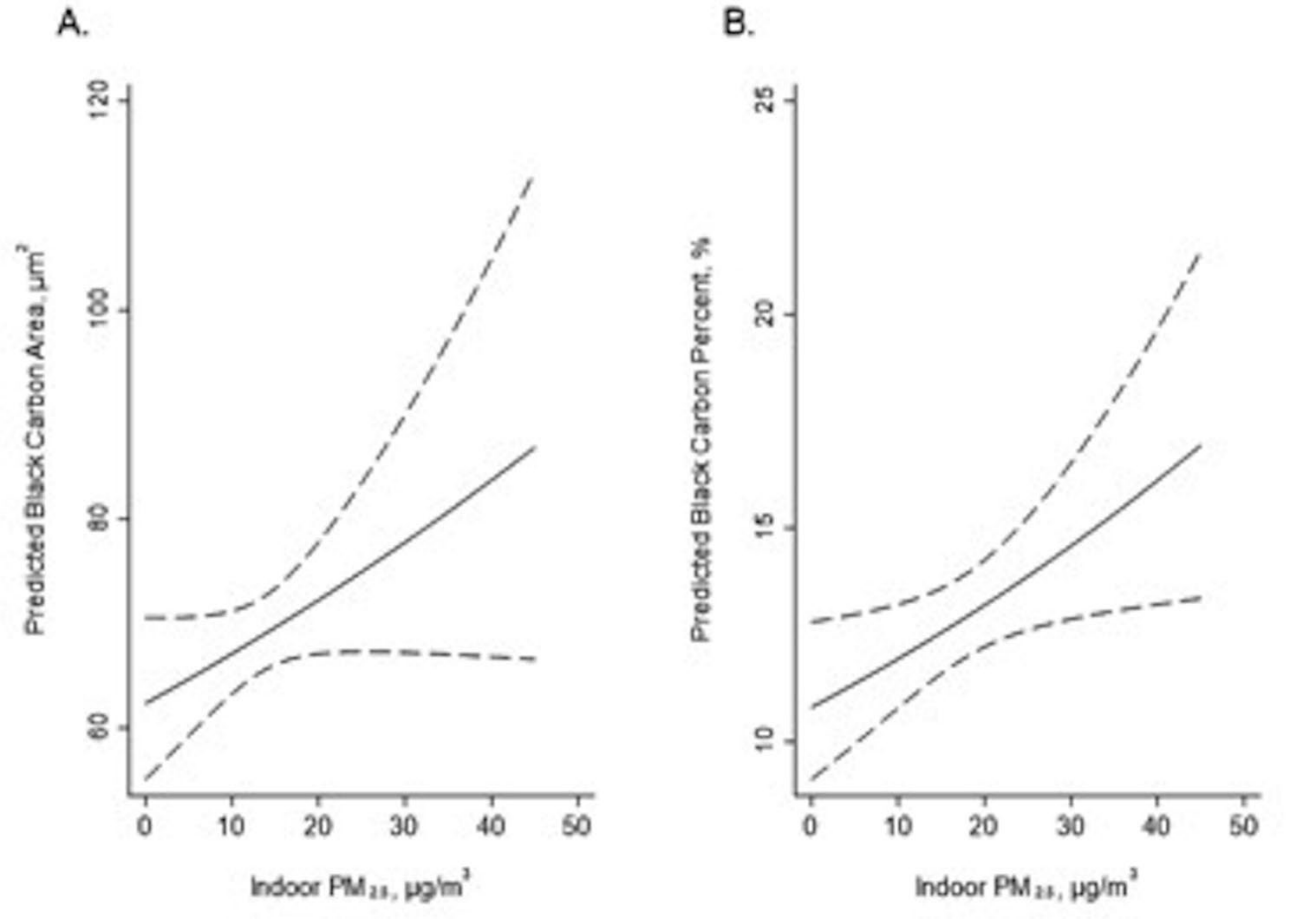
**An increase in indoor PM2.5 concentration is associated with an increase in AM black carbon levels.** The chart shows the predicted levels of (A) black carbon area and (B) black carbon percent and their 95% confidence intervals, based on multivariable regression analysis of log-transformed AM black carbon on indoor PM2.5, adjusted by batch, age, race, income, BMI, and LABA/LAMA. The predicted values were exponentiated to show the AM black carbon in original unit-scaling in y-axis

In the minimally adjusted model, adjusted for processing batch, a one SD greater (10.16 µg/m^3^) estimated indoor PM_2.5_ concentration was associated with higher BC area (β = 9.96%, 95% CI: 0.47%, 20.4%) and higher BC percent (β = 13.1%, 95% CI: 3.49%, 23.6%). When fully adjusted, indoor PM_2.5_ concentration was nominally associated with higher BC area (β = 7.78%, 95% CI: -0.96%, 17.3%, P = 0.082) (Fig. 2) and was significantly associated with a higher BC percent (β = 10.7%, 95% CI: 1.64%, 20.6%).

Urinary cotinine also was nominally associated with a higher BC area (β = 9.70%, 95% CI: -0.97%, 21.5%, P = 0.076) for a one SD rise and was statistically significantly associated with a higher BC percent (β = 10.7%, 95% CI: 2.24%, 19.9%) for a one SD rise in the fully adjusted model. There was no association of indoor NO_2_, indoor nicotine, VDGF, ambient PM_2.5_, ambient NO_2_ or ambient ozone with BC area or BC percent in either minimally adjusted or fully adjusted models (Table 2).

**Table 2:** Association of environmental exposures with black carbon area and black carbon percent

	Predicted Change (95% CI) in Black Carbon Area		Predicted Change (95% CI) in Black Carbon Percent	
	Minimally Adjusted^*^	Fully Adjusted^†^	Minimally Adjusted^*^	Fully Adjusted^†^
*Exposure*				
Indoor PM_2.5_	**10.0%** **(0.5%, 20.4%)**	7.8% (-1.0%, 17.3%)	**13.1%** **(3.5%, 23.6%)**	**10.7%** **(1.6%, 20.6%)**
Indoor NO_2_	3.5% (-4.7%, 12.3%)	2.3% (-4.5%, 9.5%)	3.4% (-4.5%, 11.9%)	1.5% (-4.5%, 7.9%)
Indoor Nicotine	4.4% (-2.6%, 12.0%)	2.7% (-2.8%, 8.6%)	5.0% (-4.5%, 15.5%)	2.9% (-5.1%, 11.6%)
Urinary Cotinine	**11.1%** **(3.1%, 19.7%)**	9.7% (-1.0%, 21.5%)	**12.5%** **(5.3%, 20.2%)**	**10.7%** **(2.2%, 19.9%)**
VGDF (yes/no)	-0.2% (-10.8%, 11.7%)	-1.9% (-12.8%, 10.4%)	2.9% (-7.7%, 14.8%)	1.0% (-9.5%, 12.7%)
Ambient PM_2.5_	1.4% (-4.2%, 7.4%)	2.7% (-3.0%, 8.6%)	-0.7% (-3.3%, 2.1%)	-0.9% (-4.4%, 2.7%)
Ambient NO_2_	1.5% (-3.1%, 6.3%)	1.2% (-3.4%, 6.0%)	-0.3% (-4.4%, 4.0%)	-1.6% (-6.4%, 3.4%)
Ambient Ozone	-0.08% (-6.7%, 7.0%)	-0.3% (-7.4%, 7.3%)	0.9% (-3.0%, 4.9%)	1.7% (-3.3%, 7.0%)

The effect estimates represent the predicted change in log AM BC, expressed in percentage change in original unit scaling, per 1 SD rise in the level (ug/m^3^) of the environmental exposure

*Adjusted for batch (first vs. second)

†Adjusted for batch (first vs. second), age, race (white vs. non-white), income (<$35,000, $35,000~$74,999, ≥$75,000, decline to answer), BMI, and LABA/LAMA use (yes vs. no)

As a sensitivity analysis, when adjusting for urinary cotinine in the fully adjusted model assessing indoor PM_2.5_ association with AM BC, a one SD greater indoor PM_2.5_ concentration showed a nominal association with AM BC (BC area: β = 4.39%, 95% CI: -4.44%, 14.3%, P = 0.335; BC percent: β = 7.71%, 95% CI: -0.34%, 16.4%, P = 0.061)

### Association of black carbon area with COPD outcomes

**Fig. 3:**
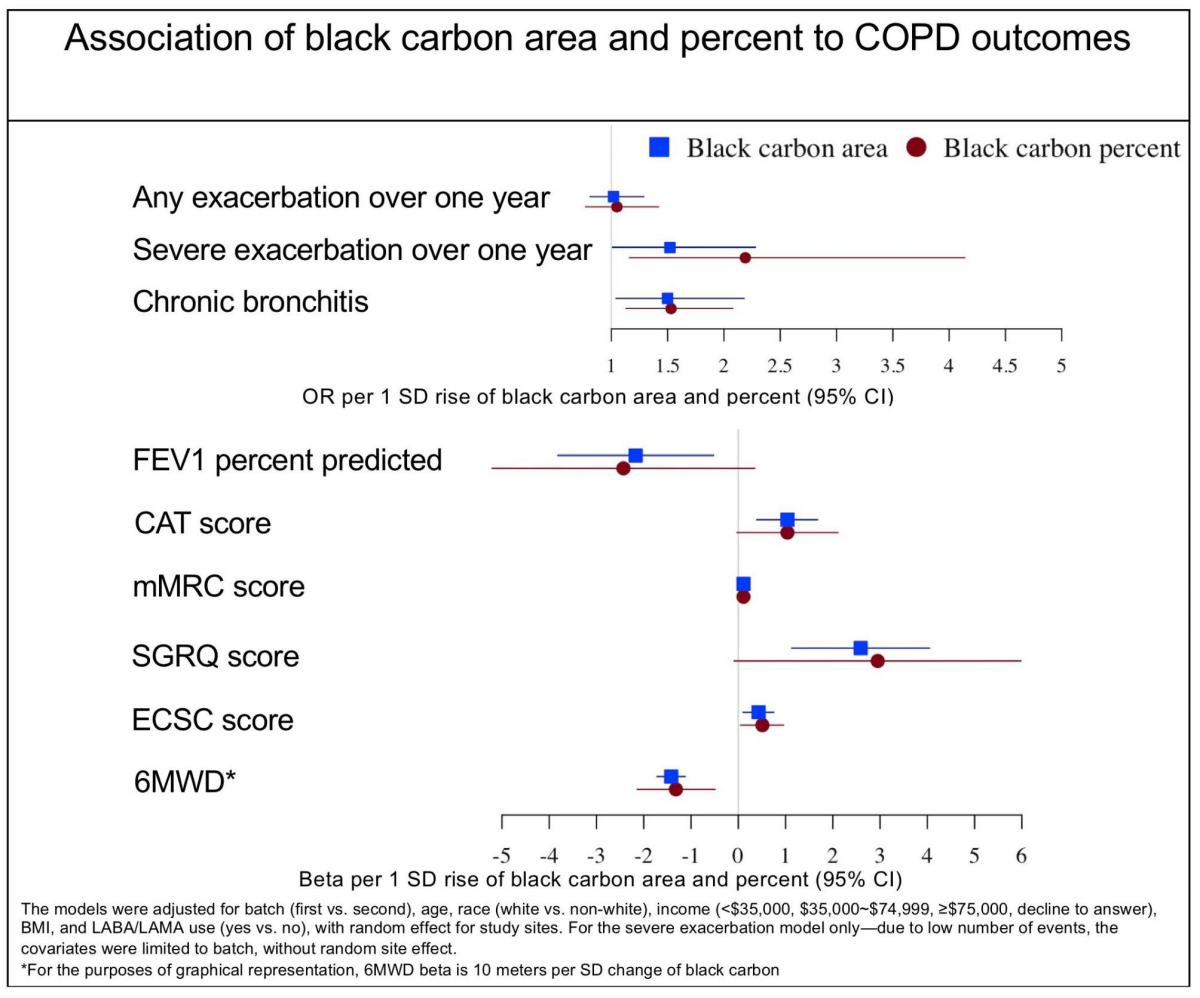
Association of black carbon area and black carbon percent with COPD outcomes

All 324 participants were included in the health outcomes analysis. After adjustment for covariates, a one SD higher (70.6 µm^2^) AM BC area was associated with lower FEV_1_% predicted (β= -2.17, 95% CI: -3.82,-0.52) and greater odds of a severe exacerbation over one year (OR = 1.52, 95% CI: 1.01, 2.28) (Fig. 3). Higher BC area was not associated with odds of any exacerbation. Higher AM BC area was associated with worse respiratory morbidity including worse CAT (β = 1.04, 95% CI: 0.39,1.68), mMRC (β = 0.11, 95% CI: 0.02–0.19), SGRQ (β = 2.59, 95% CI: 1.13–4.05), increased cough and sputum (β = 0.43, 95% CI: 0.10–0.70), shorter 6MWD (β= -14.2, 95% CI: -17.2, -11.2) and greater odds of chronic bronchitis (OR = 1.50, 95% CI: 1.04–2.18) (Table 3). In sensitivity analysis, adjusting for urinary cotinine, the results remained generally robust (e-Table 4). The association between severe exacerbation and AM BC area remained similar in magnitude and direction but was no longer statistically significant (OR = 1.47, 95% CI: 0.97, 2.22, P = 0.068). In sensitivity analysis, adjusting for FEV_1_% predicted, the results were similar (e-Table 5). AM BC area’s association with severe exacerbations was no longer statistically significant but remained similar in magnitude and direction (OR = 1.48; 95% CI: 0.95, 2.29, P = 0.08). In each sensitivity analysis, there was some suggestion of attenuation in the relationships across the outcomes, but the adjustment did not alter the conclusions.

**Table 3:** Association of black carbon area and black carbon percent with primary and secondary outcomes

	Black Carbon Area Effect Estimate (95% CI) Per 1 SD Rise	Black Carbon Percent Effect Estimate (95% CI) Per 1 SD Rise
Primary Outcomes		
FEV_1_ percent predicted	**-2.17 (-3.82, -0.52)**	-2.43 (-5.21, 0.35)
Any Exacerbation over one year, OR	1.02 (0.81, 1.29)	1.05 (0.77, 1.42)
Severe Exacerbation over one year, OR	**1.52 (1.01, 2.28)**	**2.19 (1.16, 4.14)**
Secondary Outcomes		
CAT score	**1.04 (0.39, 1.68)**	1.04 (-0.03, 2.11)
mMRC score	**0.11 (0.02, 0.19)**	**0.11 (0.03, 0.18)**
SGRQ score	**2.59 (1.13, 4.05)**	2.95 (-0.09, 5.98)
ECSC score	**0.43 (0.10, 0.75)**	**0.51 (0.05, 0.96)**
6MWD, m	**-14.17 (-17.18, -11.15)**	**-13.15 (-21.39, -4.92)**
Chronic Bronchitis, OR	**1.50 (1.04, 2.18)**	**1.53 (1.13, 2.08)**

The effect estimates represent the predicted change in each continuous COPD outcome or the odds ratio of exacerbations or chronic bronchitis per 1 SD rise in the level of AM BC measure. The models were adjusted for batch (first vs. second), age, race (white vs. non-white), income (<$35,000, $35,000~$74,999, ≥$75,000, decline to answer), BMI, and LABA/LAMA use (yes vs. no), with random effect for study sites. For the severe exacerbation model only—due to low number of events, the covariates were limited to batch, without random site effect

### Association of black carbon percent with COPD outcomes

A one SD higher (6.5 percentage point) AM BC percent was directionally associated with worse FEV_1_% predicted (β= -2.43, 95% CI: -5.21, 0.35) and greater odds of a severe exacerbation over one year (OR = 2.19, 95% CI: 1.04, 4.63). Higher BC percent was not associated with odds of any exacerbation. Higher AM BC percent was associated with worse mMRC (β = 0.11, 95% CI: 0.03,0.18), worse cough and sputum (β = 0.51, 95% CI: 0.05–0.96), shorter 6MWD (β=-13.2, 95% CI: -21.4, -4.92), and greater odds of chronic bronchitis (OR = 1.53, 95% CI: 1.13, 2.08). Higher AM BC percent was nominally associated with worse CAT and SGRQ, but these associations were not statistically significant (Table 3). Adjustment by urinary cotinine and by FEV1% predicted, in separate sensitivity analysis, did not alter the conclusion, though there was some evidence that the associations were attenuated (e-Table 4, e-Table 5).

### Interaction with smoking

There was interaction between BC area and smoking status (by urinary cotinine ≤ vs. >31.5 ng/mL) for cough and sputum (P_interaction_<0.001) and odds of chronic bronchitis (P_interaction_=0.033), such that the adverse association between AM BC area and the outcomes were stronger among current smokers than among former smokers. There was no other significant interaction for the remaining outcomes and no significant interaction between AM BC percent and smoking status for any measured outcomes (e-Table 6). Findings were similar (e-Table 7) when smoking status was characterized by self-report smoking status rather than urinary cotinine.

## Discussion

This study of former and current smokers in a multicenter cohort identified a positive association of indoor modeled PM_2.5_ and urinary cotinine with AM BC in induced sputum specimens. Additionally, AM BC area was associated with worse FEV_1_% predicted after adjusting for both previous and current cigarette smoking, as well as greater odds of a severe exacerbation. Airway macrophage BC was also associated with a shorter 6MWD, worse patient-reported outcomes and a higher likelihood of chronic bronchitis. This expands our current understanding of AM BC to serve as both a biomarker of environmental exposures as well as worse respiratory morbidity.

Compared to other studies of AM BC obtained via induced sputum, [14, 15, 32] participants from this study had a higher observed BC deposition. This may be because those studies were of children or former smokers with COPD. We have previously shown that indoor PM concentrations directly measured at participants’ residence are associated with AM BC area, [14] however in our current study utilizing indoor exposure estimates modelled on questionnaire and ambient meteorological data, we identified an association with AM BC percent and a directionally, although not statistically significant association with AM BC area in fully adjusted models. The absence of direct measurements of indoor exposures likely explains the weaker association between modeled indoor PM_2.5_ and BC area. Notably, urinary cotinine was also associated with AM BC suggesting cigarette smoke inhalation as a prominent contributing exposure. Further, the association between modeled indoor PM_2.5_ and BC was attenuated with inclusion of urinary cotinine as a covariate suggesting part, but not all, of the indoor PM which contributes to AM BC may be driven by smoking. Future studies may include never smokers to further adjudicate this possibility. Given that previous studies with measured indoor PM_2.5_ showed associations with AM BC even in non-smoking homes, [14] and the lack of association between indoor nicotine and BC in our current study, it is unlikely the association between indoor PM and BC is driven solely by cigarette smoke exposure. In terms of the role of ambient PM exposures, studies have shown that in children with asthma, ambient PM < 10 μm in aerodynamic diameter was weakly associated with AM BC [32]. This may be given BC is a reflection of combustion exposure which is predominantly in the fine particulate size. This study’s findings are consistent with prior studies of adults that demonstrated indoor – but not ambient – exposures to biomass fuel [13] or PM_2.5_ [14] are associated with increased AM BC. This may be due to the large amount of time that most adults spend indoors [33]. Lastly, we did not account for marijuana exposure which may also contribute to AM BC [34].

The impact of AM BC area on respiratory disease morbidity in COPD has been largely unexplored beyond a small single center study of former smokers with COPD demonstrating an association with increased risk of severe exacerbations and nominally worse lung function which did not identify an association with other COPD outcomes [15]. This study also identified a similar association with greater odds of a severe exacerbation. Furthermore, consistent with that prior study of former smokers with COPD [15] and a study by Kulkarni et al. of children with asthma, [32] we identified an association with worse lung function. There is no established MCID for FEV_1_% predicted, however regulators have noted a change of 5–10% of change from baseline is considered to be clinically important [35]. The BC area in our participants ranged from 20.3 to 759.8 (Fig. 1) which corresponds to an estimated 22.7% change in FEV_1_% predicted per our model in going from the lowest to the highest BC area.

Percentages of alveolar [36] and airway [37] macrophages are increased in individuals with COPD, however by randomly selecting 50 macrophages, AM content should not be influenced by the total number or percent of macrophages. Given the increase in overall macrophage count, there will be an increase in total carbon and therefore AM carbon effect may be amplified in individuals with COPD. Prior studies have typically utilized the mean carbon area per macrophage, [13–15, 20] which does not account for variation in macrophage area. However, a recent study assessing both BC area and BC percent identified stronger associations between seven day PM_2.5_ exposures and BC percent (compared to BC area) [38]. Our study also yielded stronger associations for environmental exposures and BC percent. We speculate that given BC percent intrinsically accounts for variation in macrophage size, it may be a more appropriate measure of exposures and overall BC deposition. The magnitudes of associations for both BC area and percent with clinical outcomes were similar, however the confidence intervals were wider for BC percent. These wider confidence intervals may be due to lower coefficient of variation (less dispersion around the mean) for BC percent as an independent variable—which inversely impacts the power of regression analysis—and might underpin why some associations between BC percent and clinical outcomes were not statistically significant. However, at this juncture both may be relevant biomarkers and it is premature to determine superiority of one measure compared to another.

Our study included both current and former smokers and notably, there was no effect modification by smoking status on any clinical outcomes except for the association of BC with chronic bronchitis and cough and sputum, with the deleterious effects more pronounced in current smokers. Black carbon associations with worse COPD outcomes were identified after adjusting for urinary cotinine and we therefore postulate that independent of smoking, BC serves as a useful biomarker reflecting worse disease outcomes, potentially due to direct deleterious effects on macrophage function, [15] promoting a more pro-inflammatory airway profile, [38] or increasing Th17 immunity [9].

The strength of our study lies in the comprehensive phenotype data, geographically diverse population and large sample size with former and current smokers that allowed for exploration of effect modification by smoking status. There are limitations of this study that merit discussion. Although we used a validated model of indoor exposures, we did not have direct measurements. The FEV_1_% predicted of > 35% in our cohort may not generalize to patients with more severe COPD. Additionally, we measured AM BC at a single time-point. Although the exact lifespan of human AM is unknown, 6-month exposures are most strongly associated with AM BC area [39] and in a case report following bone marrow transplantation, patients’ AMs disappeared linearly, with their life span being approximately 81 days [40]. Therefore, the 2 weeks to 1 month exposures estimates should be reflected in our BC quantification, as should the cross-sectional effect of BC on clinical outcomes. Furthermore, sputum macrophages represent a heterogenous mixture of cell age and maturation state, thus reducing any potential lifespan bias. Assessing odds of exacerbations over twelve months is longer than the reported AM lifespan however, no prior longitudinal study has identified significant within person variation of AM BC [14]. Additionally, there was a limited number of severe exacerbations which hindered our ability to adjust for confounders in our severe exacerbation model.

This study demonstrates modeled indoor PM_2.5_ and urinary cotinine are associated with increased AM BC, and increased AM BC is associated with worse lung function and greater odds of severe exacerbation, as well as worse COPD outcomes, shorter 6MWD and higher likelihood of chronic bronchitis. Therefore, beyond solely serving as a marker for increased environmental exposures, AM BC, independent of smoking status, is associated with higher disease burden, suggesting a direct deleterious effect of BC on AM in COPD. This study furthers our understanding of the influence of AM BC content on COPD morbidity.

## Electronic supplementary material

Below is the link to the electronic supplementary material.

Supplementary Material 1

## Data Availability

The data that support the findings of this study are available from SPIROMICS but restrictions apply to the availability of these data, which were used under license for the current study, and so are not publicly available. Data are however available from the authors upon reasonable request and with permission of SPIROMICS investigators

## Abbreviations

6MWD: 6 min walk distance
AM: Airway macrophages
BC: Black carbon
CAT: COPD Assessment Test
COPD: Chronic obstructive pulmonary disease
GOLD: Global Initiative for Chronic Obstructive Lung Disease
LABA/LAMA: long-acting beta-agonist or long-acting muscarinic antagonist
mMRC: modified Medical Research Council Dyspnea Scale
PM2.5: Fine (< 2.5 μm) particulate matter
NO2: Nitrogen dioxide
SGRQ: St. George’s Respiratory Questionnaire
SPIROMICS AIR: Subpopulations and Intermediate Outcome Measures In COPD Study Air Pollution Study
VGDF: vapors, gases, dusts, or fumes
