# Supplementary material for: Black carbon content in airway macrophages is associated with increased severe exacerbations and worse COPD morbidity in SPIROMICS
Source: Respir Res. 2022 Nov 14;23:310. doi: 10.1186/s12931-022-02225-0 (PMC9664618; doi:10.1186/s12931-022-02225-0)
Supplement: Supplementary file 1 — Supplementary Material 1 [file 12931_2022_2225_MOESM1_ESM.docx]

Online Supplement

# Methods

*Cytospin preparation and airway macrophage black carbon assessment*

Cytospin samples were prepared from whole sputum samples that were treated with dithiothreitol (0.1%), filtered, then stained with trypan blue and counted on a hemocytometer. Cytospin slides were generated, fixed and stained for microscopic examination of leukocyte populations. We chose acceptable quality slides based on evidence of good cell morphology (minimal cell degeneration), minimal cell debris, good quality staining, satisfactory cell dispersion and minimal squamous epithelial cell contamination. We measured AM BC from digital color images taken using the Nikon Eyepiece microscope camera (Nikon, Tokyo, Japan) at 100x magnification with oil immersion and NIS Elements Imaging Software Basic Research 5.0.

*Ambient exposure estimation*

Briefly, long-term air pollution trends were estimated from measurements collected at United States Environmental Protection Agency Air Quality System monitoring sites and other long-term monitoring locations. Partial least squares regression (PLSR) was used to create covariates that combine geographic variables including land use, distance to roadways, vegetative cover, impervious surface cover, traffic data, population and elevation. Using these PLSR covariates and spatial smoothing, spatially varying coefficients for the time trends were estimated, predicting the long-term mean and scale of temporal variation based on point-specific characteristics. The model benefits from spatially rich supplemental monitoring data that are leveraged to estimate the spatially varying coefficients and determine the structure of the spatio- temporal residual field. Furthermore, measured ambient concentrations from a sample of SPIROMICS participants were incorporated into the prediction model.

*Indoor exposure estimation*

Briefly, models used questionnaire data (i.e., socioeconomic, behavioral, residential characteristics) and meteorological and ambient-pollutant concentration data to estimate indoor pollutant concentrations. The model for predicting PM2.5 concentration included 3 questions related to smoking habits. Similar to the ambient models, measured indoor concentrations from a sample of SPIROMICS participants were incorporated into the prediction model.

*Multivariable Regression of AM Black Carbon on Indoor Pollutant Exposures*

To assess the relationship between AM black carbon and indoor PM2.5, linear regression of BC area or BC percent was run on indoor PM2.5 using generalized linear mixed model with random effect for study sites. The models were minimally adjusted for batch (first vs. second) and fully adjusted for batch, age, race (white vs. non-white), income (<$35,000,

$35,000~$74,999, ≥$75,000, decline to answer), BMI, and long-acting beta-agonist/long-acting muscarinic antagonists (LABA/LAMA) use. The covariates were chosen based on both

theoretical considerations from prior literature and empirical assessment for potential confounding based on bivariate associations (P<0.2) with AM black carbon and indoor pollutant measures. Other potential confounders considered but not included were sex (male vs female), education (high school or less vs more than high school), pack-years, inhaled corticosteroid (ICS), and ICS/LABA/LAMA, as they did not meet the criteria of potential confounding based on bivariate associations (P<0.2). Sensitivity analysis was conducted using urinary cotinine, which is a continuous measure of quantity smoked and second-hand smoke exposure in the prior 3-4 days, as a covariate in the fully adjusted models. As secondary analysis, other environmental exposures (indoor NO2, indoor nicotine, urinary cotinine, VGDF exposure (yes/no), ambient PM2.5, ambient NO2, and ambient ozone) were assessed for their associations with BC area or BC percent one at a time using the same regression approach. Linearity assumption was checked using restricted cubic spline model and the normality of continuous residuals assessed using normal quantile plot; the consistency of parameter estimates across different variable transformations were examined. BC measures, as dependent variables, were modeled continuously and log-transformed. Indoor and ambient pollutant measures were modeled continuously.

*Multivariable Regression of COPD Outcomes on AM Black Carbon*

To assess the relationship between COPD outcomes and AM BC, linear regression of continuous COPD outcomes and logistic regression of any or severe exacerbations and chronic bronchitis was run on BC area or BC percent using generalized linear mixed model with random effect for study sites. Based on the covariate selection approach described above, all models were adjusted for batch, age, sex, race (white vs non-white), income (<$35,000, $35,000~$74,999,

≥$75,000, decline to answer), BMI, and LABA/LAMA use. However, for severe exacerbation model only, due to low number of reported events, the covariates were limited to batch without random site effect. In sensitivity analysis, urinary cotinine and FEV1 % predicted were additionally adjusted as a covariate one at a time. The primary exposure was BC area and the secondary exposure BC percent. As exposures (not as outcomes) neither BC variable required log-transformation based on linearity and normality diagnostics, and thus for the ease of interpretation, original unit scaling was kept. Robust standard error was used in all models. The current smokers were defined as those whose urinary cotinine levels were >31.5 ng/mL. To assess whether the association between COPD outcomes and AM BC vary by smoking status (current vs. former smokers defined by urinary cotinine cut-off), a two-way interaction model was run by adding the interaction between dichotomous smoking status and BC area or BC percent to the main model. Additionally, stratified analyses by smoking status were performed using fully adjusted models. For any and severe exacerbation models, due to the low number of reported events within each subgroup, covariate list were modified in stratified analyses. For the any exacerbation model, the covariates were limited to batch and LABA/LAMA (yes vs. no)— both of which were selected based on each variable’s bivariate association with any exacerbation and with AM BC. For the severe exacerbation model, the stratified analysis among former smokers was not performed because the number of reported events was less than 5—the minimum rule-of-thumb number that we used to decide whether to perform the analysis or not. The stratified analysis among current smokers was performed for the severe exacerbation model, and the model was adjusted by batch only. For the 2-way interaction models, the any

exacerbation analysis was run fully adjusted, while the severe exacerbation analysis was not performed due to low number of events and the required number of predictors to include.

# Tables

**e-Table 1: Institutional Review Board (IRB) committee name and approval number at each SPIROMICS center**

| **SPIROMICS Center** | **Institutional Review Board Committee Name** | **Approval Number** |
| --- | --- | --- |
| Columbia University | Columbia University IRB 2 | IRB-AAAE9315 |
| University of Iowa | University of Iowa Hawk IRB-01 | 201308719 |
| Johns Hopkins University | Johns Hopkins IRB-5 | NA_00035701 / CR00018131 |
| University of California Los Angeles | UCLA Medical IRB 1 (MIRB1). | 10001740 |
| University of Michigan | University of Michigan IRBMED B1 Board | HUM00036346 |
| National Jewish Health | National Jewish Health Institutional Review board | 19970 |
| University of California San Francisco | UCSF IRB Parnassus Panel | 10-03169 |
| Temple University | Temple University IRB A2 | 21416 |
| University of Alabama at Birmingham | U of Alabama at Birmingham IRB #2 | 120906004 |
| University of Illinois | University of Illinois Institutional Review Board (IRB) #3 | 2013-0939 |
| University of Utah | University of Utah IRB Panel Review Board 5 | 00027298 |
| Wake Forest University | Wake Forest University IRB #5 | 00012805 |

**e-Table 2. Participants including in our analysis with black carbon quantification compared to all SPIROMICS participants with an FEV1 percent predicted >35%**

|  | Black carbon study  N = 324 | SPIROMICS  cohort with FEV1%  predicted >35%  N = 2,487 | P-value |
| --- | --- | --- | --- |
| Age, yrs | 63.58 ± 8.78 | 63.54 ± 9.09 | 0.943 |
| Sex, n (% female) | 134 (41.4%) | 1155 (46.4%) | 0.084 |
| Race, n (% white) | 260 (80.7%) | 1897 (76.7%) | 0.107 |
| Education, n (% > HS grad) | 223 (69.0%) | 1539 (62.2%) | 0.016 |
| Annual Household Income |  |  | 0.122 |
| <$35,000 | 116 (35.8%) | 946 (38.0%) |  |
| $35,000 - $74,999 | 101 (31.2%) | 663 (26.7%) |  |
| ≥$35,000 | 62 (19.1%) | 431 (17.3%) |  |
| Decline to answer | 45 (13.9%) | 447 (18.0%) |  |
| Body Mass Index | 28.50 ± 5.07 | 28.12 ± 5.24 | 0.220 |
| Medication, n (% LABA/LAMA) | 87 (27.1%) | 930 (37.7%) | <0.001 |
| Urinary Cotinine, ng/ mL | 597.70 ± 851.23 | 571.08 ± 812.96 | 0.584 |
| Pack-Years, yrs | 47.76 ± 22.46 | 48.92 ± 27.16 | 0.463 |
| FEV1 percent predicted | 84.30 ± 18.96 | 78.37 ± 22.44 | <0.001 |
| Current Smoker, n (% yes) | 143 (44.4%) | 1037 (42.3%) | 0.466 |
| ICS, n (% yes) | 69 (21.5%) | 785 (31.8%) | <0.001 |
| OCS, n (% yes) | 2 (0.6%) | 35 (1.4%) | 0.241 |
| CAT | 12.31 ± 7.72 | 13.45 ± 8.11 | 0.018 |
| mMRC | 0.83 ± 0.82 | 0.96 ± 0.93 | 0.012 |
| SGRQ | 28.26 ± 19.34 | 31.32 ± 20.06 | 0.013 |
| ECSC | 9.16 ± 3.49 | 9.20 ± 3.47 | 0.825 |
| 6MWD, meters | 432.99 ± 101.29 | 407.57 ± 107.01 | <0.001 |
| Any exacerbations in the prior 12mo, n (% yes) | 51 (16.1%) | 548 (22.3%) | 0.012 |
| Severe exacerbations in the prior 12mo, n (% yes) | 22 (6.9%) | 243 (9.9%) | 0.092 |
| Any exacerbations over 12mo follow-up, n (% yes) | 36 (11.9%) | 408 (17.7%) | 0.011 |
| Severe exacerbations over 12mo follow-up, n (% yes) | 8 (2.6%) | 129 (5.6%) | 0.030 |

**e-Table 3. Participants included in our analysis with non-missing ambient or indoor pollutant data compared to those with missing ambient or indoor pollutant data.**

|  | Participants with pollutant data  N = 285 | Participants without pollutant data  N = 39 | P-  value |
| --- | --- | --- | --- |
| Age, yrs | 63.81 ± 8.77 | 61.87 ± 8.73 | 0.196 |
| Sex, n (% female) | 119 (41.8%) | 15 (38.5%) | 0.695 |
| Race, n (% white) | 228 (80.6%) | 32 (82.1%) | 0.825 |
| Education, n (% > HS grad) | 204 (71.6%) | 19 (50.0%) | 0.007 |
| Annual Household Income |  |  | 0.041 |
| <$35,000 | 97 (34.0%) | 19 (48.7%) |  |
| $35,000 - $74,999 | 89 (31.2%) | 12 (30.8%) |  |
| ≥$75,000 | 54 (18.9%) | 8 (20.5%) |  |
| Decline to answer | 45 (15.8%) | 0 (0.0%) |  |
| Body Mass Index | 28.64 ± 5.00 | 27.48 ± 5.51 | 0.180 |
| Medication, n (% LABA/LAMA) | 77 (27.3%) | 10 (25.6%) | 0.827 |
| Urinary Cotinine, ng/ mL | 584.84 ± 849.46 | 690.32 ± 869.32 | 0.469 |
| Pack-Years, yrs | 47.53 ± 22.26 | 49.40 ± 24.14 | 0.628 |
| FEV1 percent predicted | 84.54 ± 19.25 | 82.57 ± 16.85 | 0.544 |
| Percent Emphysema <-950 HU | 3.97 ± 4.67 | 3.31 ± 4.36 | 0.399 |
| Current Smoker, n (% yes) | 122 (43.1%) | 21 (53.8%) | 0.206 |
| ICS, n (% yes) | 64 (22.7%) | 5 (12.8%) | 0.159 |
| OCS, n (% yes) | 2 (0.7%) | 38 (100.0%) | 0.602 |
| CAT | 12.07 ± 7.43 | 14.03 ± 9.49 | 0.138 |
| mMRC | 0.81 ± 0.80 | 0.97 ± 0.96 | 0.227 |
| SGRQ | 27.53 ± 18.61 | 33.29 ± 23.47 | 0.086 |
| ECSC | 9.14 ± 3.46 | 9.26 ± 3.77 | 0.848 |
| 6MWD, meters | 431.56 ± 99.78 | 443.87 ± 113.00 | 0.488 |
| Total exacerbations in the prior 12mo, n (% yes) | 44 (15.8%) | 7 (17.9%) | 0.736 |
| Severe exacerbations in the prior 12mo, n (% yes) | 20 (7.2%) | 2 (5.1%) | 0.634 |
| Any exacerbations over 12mo follow-up, n (% yes) | 30 (11.3%) | 6 (15.8%) | 0.426 |
| Severe exacerbations over 12mo follow-up, n (% yes) | 7 (2.6%) | 1 (2.6%) | 0.997 |

**e-Table 4. Sensitivity analysis of association of black carbon area and black carbon percent with primary and secondary outcomes, with urinary cotinine added as a covariate**

|  | Black Carbon Area Effect Estimate (95% CI) Per 1 SD Rise | Black Carbon Percent Effect Estimate (95% CI) Per 1 SD Rise |
| --- | --- | --- |
| Primary Outcomes |  |  |
| FEV1 percent predicted | **-2.02 (-3.84, -0.19)** | -2.09 (-4.63, 0.44) |
| Any Exacerbation over one year, OR | 1.05 (0.86, 1.27) | 1.10 (0.86, 1.42) |
| Severe Exacerbation over one  year, OR | 1.47 (0.97, 2.22) | **2.15 (1.12, 4.10)** |
| Secondary Outcomes |  |  |
| CAT | **0.92 (0.09, 1.75)** | 0.78 (-0.22, 1.79) |
| mMRC | **0.10 (0.01, 0.19)** | **0.09 (0.03, 0.15)** |
| SGRQ | **2.30 (0.50, 4.10)** | 2.26 (-0.61, 5.13) |
| Cough & Sputum | **0.37 (0.08, 0.67)** | 0.38 (-0.01, 0.77) |
| 6MWD | **-14.10 (-16.59, -11.62)** | **-12.13 (-19.19, -5.07)** |
| Chronic Bronchitis, OR | **1.42 (1.06, 1.91)** | **1.43 (1.10, 1.86)** |

The effect estimates represent the predicted change in each continuous COPD outcome, or the hazard ratio of exacerbations, or the odds ratio of chronic bronchitis per 1 SD rise in the level of AM black carbon measure. The models were adjusted for urinary cotinine, batch (first vs. second), age, race (white vs. non-white), income (<$35,000, $35,000~$74,999, ≥$75,000, decline to answer), BMI, and LABA/LAMA use (yes vs. no), with random effect for study sites. For the severe exacerbation model only—due to low number of events, the covariates were limited to batch, without random site effect.

**e-Table 5. Sensitivity analysis of association of black carbon area and black carbon percent with primary and secondary outcomes, with FEV1 percent predicted added as a covariate**

|  | Black Carbon Area Effect Estimate (95% CI) Per 1 SD Rise | Black Carbon Percent Effect Estimate (95% CI) Per 1 SD Rise |
| --- | --- | --- |
| Primary Outcomes |  |  |
| Any Exacerbation over one year,  OR | 1.00 (0.81, 1.23) | 1.01 (0.76, 1.34) |
| Severe Exacerbation over one year, OR | 1.48 (0.95, 2.29) | **2.21 (1.06, 4.61)** |
| Secondary Outcomes |  |  |
| CAT | **0.84 (0.24, 1.45)** | 0.82 (-0.29, 1.94) |
| mMRC | **0.09 (0.02, 0.17)** | **0.09 (0.02, 0.16)** |
| SGRQ | **2.13 (0.77, 3.49)** | 2.44 (-0.43, 5.32) |
| Cough & Sputum | 0.36 (-0.01, 0.74) | 0.44 (-0.03, 0.90) |
| 6MWD | **-13.17 (-16.84, -9.50)** | **-12.15 (-20.98, -3.31)** |
| Chronic Bronchitis, OR | 1.46 (0.97, 2.18) | **1.47 (1.07, 2.03)** |

The effect estimates represent the predicted change in each continuous COPD outcome, or the hazard ratio of exacerbations, or the odds ratio of chronic bronchitis per 1 SD rise in the level of AM black carbo measure. The models were adjusted for FEV1 % predicted, batch (first vs. second), age, race (white vs. non-white), income (<$35,000, $35,000~$74,999, ≥$75,000, decline to answer), BMI, and LABA/LAMA use (yes vs. no), with random effect for study sites. For the severe exacerbation model only—due to low number of events, the covariates were limited to batch, without random site effect.

**e-Table 6. Stratified analysis by smoking status of black carbon area and black carbon percent with primary and secondary outcomes**

|  | Black Carbon Area Effect Estimate (95% CI) Per 1 SD Rise | | Black Carbon Percent Effect Estimate (95% CI) Per 1 SD Rise | |
| --- | --- | --- | --- | --- |
|  | Former Smokers (urinary cotinine  ≤31.5 ng/mL) N=160 | Current Smokers (urinary cotinine  >31.5 ng/mL) N=160 | Former Smokers (urinary cotinine  ≤31.5 ng/mL) N=160 | Current Smokers (urinary cotinine  >31.5 ng/mL) N=160 |
| Primary Outcomes |  |  |  |  |
| FEV1 percent predicted | **-2.22** | -1.81 | **-2.01** | -2.43 |
|  | **(-3.73, -0.71)** | (-4.05, 0.44) | **(-3.66, -0.36)** | (-5.91, 1.05) |
| Any Exacerbation over | 0.99 | 1.12 | 1.01 | 1.22 |
| one year, OR | (0.47, 2.09) | (0.62, 2.02) | (0.45, 2.25) | (0.74, 2.03) |
| Severe Exacerbation over | - | **1.87** | - | **2.99** |
| one year, OR |  | **(1.28, 2.73)** |  | **(1.15, 7.72)** |
| Secondary Outcomes |  |  |  |  |
| CAT | 0.71 | **0.92** | 0.87 | 0.50 |
|  | (-0.29, 1.70) | **(0.04, 1.79)** | (-0.21, 1.95) | (-0.70, 1.70) |
| mMRC | **0.14** | 0.07 | **0.11** | 0.06 |
|  | **(0.07, 0.21)** | (-0.04, 0.19) | **(0.08, 0.15)** | (-0.06, 0.18) |
| SGRQ | 0.55 | **2.52** | -0.004 | 2.75 |
|  | (-1.50, 2.60) | **(0.97, 4.07)** | (-2.98, 2.97) | (-0.53, 6.04) |
| Cough & Sputum | -0.06 | **0.55*** | 0.26 | **0.45** |
|  | (-0.43, 0.31) | **(0.29, 0.80)** | (-0.24, 0.77) | **(0.08, 0.82)** |
| 6MWD | -19.18 | **-9.98** | **-18.23** | -5.30 |
|  | (-39.35, 1.00) | **(-17.52, -2.44)** | **(-33.95, -2.52)** | (-14.87, 4.26) |
| Chronic Bronchitis, OR | 1.14 | **2.07*** | **1.38** | 1.54 |
|  | (0.77, 1.68) | **(1.19, 3.60)** | **(1.12, 1.70)** | (0.94, 2.52) |

*Indicates statistical significant interaction between dichotomous smoking status (former vs. current smoker, as defined by urinary cotinine ≤ vs. > 31.5 ng/mL) and AM black carbon measure on COPD outcomes. The effect estimates represent the predicted change in each continuous COPD outcome, or the hazard ratio of exacerbations, or the odds ratio of chronic bronchitis per 1 SD rise in the level of AM black carbon measure within each subgroup. The model was fully adjusted. For any and severe exacerbation models, due to low number of events within subgroups, covariate list were modified; the covariates in any exacerbation model was limited to batch and LABA/LAMA (yes vs. no)—based on the variable’s bivariate association with any severe exacerbation and with AM BC, while the covariates in severe exacerbation model was limited to batch and without random site effect. For the severe exacerbation model, the stratified analysis within former smokers was not performed due to the fact that the number of severe exacerbation events within this subgroup was less than 5—a minimum rule of thumb number that we used to decide whether to perform the analysis or not. For the 2-way interaction models, the any exacerbation analysis was run fully adjusted, while the severe exacerbation analysis was not performed due to low number of events and the required number of predictors to include.

**e-Table 7. Stratified analysis by smoking status (as defined by self-report) of black carbon area and black carbon percent with primary and secondary outcomes**

|  | Black Carbon Area  Effect Estimate (95% CI)  Per 1 SD Increase | | Black Carbon Percent  Effect Estimate (95% CI)  Per 1 SD Increase | |
| --- | --- | --- | --- | --- |
|  | Former Smokers (self-reported)  N=179 | Current Smokers  (self-reported)  N=143 | Former Smokers (self-reported)  N=179 | Current Smokers  (self-reported)  N=143 |
| Primary Outcomes |  |  |  |  |
| FEV1 percent predicted | **-1.83**  **(-3.60, -0.05)** | **-2.29**  **(-4.10, -0.49)** | **-2.13**  **(-4.10, -0.15)** | -2.71  (-6.27, 0.86) |
| Time to first event:  Any exacerbations, HR | 0.89  (0.59, 1.34) | **1.19**  **(1.03, 1.37)** | 1.01  (0.57, 1.78) | 1.20  (0.88, 1.64) |
| Time to first event:  Severe exacerbations, HR | 1.01  (0.73, 1.38) | 1.11  (0.78, 1.59) | 1.21  (0.85, 1.71) | 1.43  (0.61, 3.36) |
| Secondary Outcomes |  |  |  |  |
| CAT | 0.52  (-0.07, 1.12) | **0.99**  **(0.004, 1.98)** | 0.84  (-0.23, 1.92) | 0.35  (-1.18, 1.87) |
| mMRC | 0.06  (-0.02, 0.14) | **0.13***  **(0.04, 0.22)** | **0.09**  **(0.03, 0.14)** | **0.11**  **(0.001, 0.21)** |
| SGRQ | 0.82  (-1.21, 2.85) | **2.70**  **(0.76, 4.64)** | 0.17  (-3.21, 3.56) | 2.91  (-1.02, 6.84) |
| Cough & Sputum | 0.11  (-0.46, 0.67) | **0.48**  **(0.20, 0.76)** | 0.19  (-0.32, 0.69) | **0.40**  **(0.01, 0.79)** |
| 6MWD | -9.49  (-21.94, 2.95) | **-12.60**  **(-17.54, -7.65)** | -11.21  (-28.89, 6.48) | -4.15  (-14.39, 6.09) |
| Chronic Bronchitis, OR | 1.24  (0.90, 1.71) | 1.71  (0.99, 2.93) | **1.30**  **(1.04, 1.61)** | 1.37  (0.84, 2.24) |

*Indicates statistical significant interaction between dichotomous smoking status (as defined by urinary cotinine level) and AM black carbon measure on COPD outcomes. The effect estimates represent the predicted change in each continuous COPD outcome, or the hazard ratio of exacerbations, or the odds ratio of chronic bronchitis per 1 SD increase in the level of AM black carbon measure within each subgroup. The model was fully adjusted. For the severe exacerbation model only—due to few events within subgroup, the covariates were limited to BMI, without random effect for study sites.

**e-Figure 1. Histograms showing distribution of black carbon area and black carbon percent among current and former smokers**

**e-Figure 2. Example of airway macrophages with (A) high and (B) low carbon black**
